# Hitchhiking the high seas: Global genomics of rafting crabs

**DOI:** 10.1002/ece3.4694

**Published:** 2019-01-23

**Authors:** Joseph B. Pfaller, Adam C. Payton, Karen A. Bjorndal, Alan B. Bolten, Stuart F. McDaniel

**Affiliations:** ^1^ Department of Biology, Archie Carr Center for Sea Turtle Research University of Florida Gainesville Florida; ^2^ Caretta Research Project Savannah Georgia; ^3^ Department of Biology University of Florida Gainesville Florida

**Keywords:** dispersal, phylogeography, *Planes*, population structure, rafting

## Abstract

Population differentiation and diversification depend in large part on the ability and propensity of organisms to successfully disperse. However, our understanding of these processes in organisms with high dispersal ability is biased by the limited genetic resolution offered by traditional genotypic markers. Many neustonic animals disperse not only as pelagic larvae, but also as juveniles and adults while drifting or rafting at the surface of the open ocean. In theory, the heightened dispersal ability of these animals should limit opportunities for species diversification and population differentiation. To test these predictions, we used next‐generation sequencing of genomewide restriction‐site‐associated DNA tags (RADseq) and traditional mitochondrial DNA sequencing, to investigate the species‐level relationships and global population structure of *Planes *crabs collected from oceanic flotsam and sea turtles. Our results indicate that species diversity in this clade is low—likely three closely related species—with no evidence of cryptic or undescribed species. Moreover, our results indicate weak population differentiation among widely separated aggregations with genetic indices showing only subtle genetic discontinuities across all oceans of the world (RADseq *F*_ST_ = 0.08–0.16). The results of this study provide unprecedented resolution of the systematics and global biogeography of this group and contribute valuable information to our understanding of how theoretical dispersal potential relates to actual population differentiation and diversification among marine organisms. Moreover, these results demonstrate the limitations of single gene analyses and the value of genomic‐level resolution for estimating contemporary population structure in organisms with large, highly connected populations.

## 1 INTRODUCTION

Population differentiation and ultimately diversification depend in large part on the ability and propensity of organisms to successfully disperse (Palumbi, 1994, 2003 ). Organisms with high dispersal ability are predicted to have high levels of gene flow among distant populations, leading to limited population structure and reduced opportunities for diversification (Avise, 2000; Goetze, 2005; but see Fraser, Banks, & Waters, 2015). Conversely, organisms with weak dispersal ability are predicted to have low levels of gene flow, high degrees of population structure, and elevated rates of diversification (Avise, 2000; Palumbi, 1994, 2003 ). Thus, understanding the consequences of dispersal ability is fundamental to our understanding of population and community ecology, as well as the origin and maintenance of biological diversity (Lenormand, 2002; McPeek & Holt, 1992; Treml, Ford, Black, & Swearer, 2015).

Among marine animals, pelagic larval duration (PLD) plays a widely recognized role in the dispersal and connectivity of populations (Faurby & Barber, 2012). Because adults of many marine animals are nondispersive—often benthic and sessile or sedentary—dispersal is primarily restricted by the vagility of pelagic larvae. As a result, PLD is often correlated with the geographic range and degree of population differentiation (e.g., *F*_ST_) of a species (Cowen, 2000; Cowen & Sponaugle, 2009, but see Bradbury, Laurel, Snelgrove, Bentzen, & Campana, 2008; Weersing & Toonen, 2009). However, the communities of animals associated with the ocean's air–water interface (termed Neuston: Naumann, 1917 via Marshall & Burchardt, 2005) also disperse as juveniles and adults while drifting or rafting at the surface of the open ocean. In these animals, dispersal by pelagic larvae (if present) is augmented by the dispersal potential of adults and juveniles, which can use large ocean currents to disperse across ocean basins and perhaps further (Thiel & Haye, 2006). In theory, the heightened dispersal ability of these animals should lead to wide geographic ranges, limited population structure, and reduced diversification. However, unlike many members of benthic communities, we know far less about population differentiation and diversification within the neustonic community.

Marine organisms with large populations and high dispersal ability pose challenges for estimating population connectivity and structure because tracking dispersal of individuals within the vast ocean is often logistically impossible. Genetic data provide a tool to assess population connectivity at large spatial scales (Hedgecock, Barber, & Edmands, 2007; Lowe & Allendorf, 2010). Because the detection of significant population structure provides clear evidence for differentiation among populations with low connectivity, the literature is biased toward positive examples of population structure (Hedgecock et al., 2007). However, when population connectivity is high and populations are large (e.g., in neustonic animals), it is difficult to detect population structure with limited genetic resolution offered by traditional genotypic markers (Benestan et al., 2015; Goetze, 2005; McCormack, Hird, Zellmer, Carstens, & Brumfield, 2013). Recent advances in high‐throughput sequencing technologies have allowed genomewide genetic variation to be incorporated in population genetic analyses of nonmodel organisms (Reitzel, Herrera, Layden, Martindale, & Shank, 2013), providing unprecedented genetic resolution of the population biology and connectivity of previously enigmatic groups of organisms.

*Planes *crabs are common and conspicuous members of the neustonic community throughout the temperate and tropical oceans of the world (Chace, 1951). Three species currently are recognized as follows: *Planes minutus *(N. Atlantic and Mediterranean), *Planes major* (worldwide, except N. Atlantic), and *Planes marinus* (worldwide, except N. Atlantic) (Chace, 1951; Ng, Guinot, & Davie, 2008). While *Pl. marinus* is morphologically distinct, *Pl. minutus *and *Pl. major *show overlapping trait distributions in supposedly diagnostic traits (Chace, 1951). Recent phylogenetic analyses of the family Grapsidae suggest that the genus *Planes *is actually paraphyletic due to the well‐supported inclusion of a fourth putative species, *Pachygrapsus laevimanus *(Ip, Schubart, Tsang, & Chu, 2015; Schubart, 2011), which is an intertidal species found across a narrow band of the South Pacific from Australia to Rapa Island (Poupin, Davie, & Cexus, 2005). Unlike intertidal grapsid crabs, which disperse almost exclusively during the pelagic larvae stage (Anger, 1995), *Planes *disperse as juveniles and adults while rafting on surface‐drifting oceanic debris or flotsam, and as facultative symbionts of oceanic‐stage sea turtles, frequently inhabiting the pocket above the turtle's tail (Chace, 1951; Pfaller et al., 2014). *Planes *and *Pa. laevimanus *crabs therefore provide an opportunity to test the prediction that elevated dispersal potential limits species diversity and decreases population structure.

Traditional single gene analyses of intertidal grapsid crabs show weak genetic differentiation across wide latitudinal gradients, but limited evidence for transoceanic gene flow, indicating that large ocean basins represent significant barriers to pelagic larval dispersal (Cassone & Boulding, 2006; Schubart, Cuesta, & Felder, 2005). Moreover, restricted transoceanic gene flow between sister species has been identified as a potential mechanism leading to diversification within the family Grapsidae (Schubart, 2011). For *Planes*, the ability to disperse as pelagic larvae and as adults associated with oceanic flotsam and sea turtles should facilitate transoceanic genetic exchange, limiting both species diversity within this group of crabs and intraspecific genetic differentiation among widely separated populations. To test this hypothesis, we conducted an analysis of the global species diversity and population‐level differentiation using next‐generation sequencing of genomewide restriction‐site‐associated DNA tags (RADseq) and traditional mitochondrial DNA (mtDNA) sequencing, to address three main questions. At the species level: (a) Is species diversity low with no evidence of cryptic species? At the population level: (b) Is population differentiation weak among widely separated aggregations? (c) If genetic discontinuities exist, where are there biogeographic corridors and barriers to rafting dispersal at a global scale?

## 2 METHODS

### 2.1 Taxon sampling and justification

**Figure 1:**
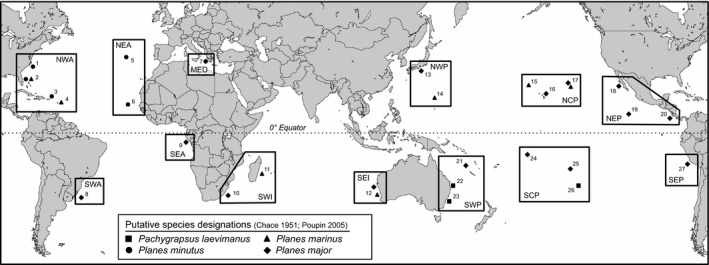
Map showing collecting locations in 13 ocean regions (and regional abbreviations) for each putative species. Black boxes indicate how sampling sites were grouped into broad ocean regions and do not represent biogeographic boundaries. *Notes*. NWA, northwest Atlantic; NEA, northeast Atlantic; MED, Mediterranean Sea; SWA, southwest Atlantic; SEA, southeast Atlantic; SWI, southwest Indian; SEI, southeast Indian; NWP, northwest Pacific; NCP, north central Pacific; NEP, northeast Pacific; SWP, southwest Pacific; SCP, south central Pacific; SEP, southeast Pacific. 1, North Carolina, USA; 2, Florida, USA; 3, Puerto Rico; 4, St. Martin; 5, Azores; 6, Cape Verde; 7, Greece; 8, Brazil; 9, Gabon; 10, South Africa; 11, Madagascar; 12, Western Australia; 13, Japan; 14, Northern Mariana Islands; 15, northwest of Hawaii; 16, Hawaii; 17, northeast of Hawaii (Great Pacific Garbage Patch); 18, Mexico; 19, Clipperton Island; 20, Costa Rica; 21, New Caledonia; 22, Queensland, Australia; 23, New South Wales, Australia; 24, Samoa; 25, Moorea; 26, Rapa Island; 27, Peru

Specimens were collected from 27 sites within 13 broad ocean regions corresponding to the east and west sides of each major ocean gyre, the central Pacific, and the Mediterranean Sea (Figure 1; sampling regions were not based on known biogeographic boundaries). Each specimen was given an a priori species designation based on external morphology, habitat, and/or geography following Chace (1951) and Poupin et al. (2005) (see Appendix S1 for details). *Pachygrapsus laevimanus *specimens were collected intertidally among rocks at three sites across its known range (Poupin et al., 2005). *Planes *specimens were collected from surface‐drifting oceanic debris and sea turtles (*Caretta caretta*, *Chelonia mydas*, *Eretmochelys imbricata,* and *Lepidochelys olivacea*) at 24 sites within all 13 ocean regions. Where applicable for each putative species, 1–5 individuals were obtained from each site. For *Pl. minutus *and *Pl. major*, larger samples (>10 individuals) were collected when possible (Table 1).

**Table 1:** Sample sizes by putative species, region, habitat, and genetic analysis

Putative species designations (Chace, 1951; Poupin et al., 2005 )	Region^b^	Habitat^c^	Number of individuals^a^			
			*COI *only	*RAD *only	*COI *and *RAD*	Total
*Pachygrapsus laevimanus*	SWP	Intertidal	0	2	4	6
*Pa. laevimanus* d	SCP	Intertidal	2	0	0	2
*Planes marinus*	NWA	Turtles/Flotsam	0	0	3	3
*Pl. marinus*	SWI	Flotsam	0	0	1	1
*Pl. marinus*	SEI	Flotsam	0	0	2	2
*Pl. marinus*	NWP	Flotsam	0	0	1	1
*Pl. marinus*	NCP	Flotsam	3	3	1	7
*Planes minutus*	NWA	Turtles	4	11	7	22
*Pl. minutus*	NEA	Turtles	4	16	13	33
*Pl. minutus*	MED	Turtles	1	4	1	6
*Planes major*	SWA	Turtles	0	0	2	2
*Pl. major*	SEA	Turtle	1	0	1	2
*Pl. major*	SWI	Turtles/Flotsam	0	6	1	7
*Pl. major*	SEI	Flotsam	0	0	1	1
*Pl. major*	NWP	Turtles	1	18	3	22
*Pl. major*	NCP	Turtles/Flotsam	3	7	6	16
*Pl. major*	NEP	Turtles/Flotsam	0	7	7	14
*Pl. major*	SWP	Flotsam	0	0	2	2
*Pl. major*	SCP	Turtles/Flotsam	1	0	3	4
*Pl. major*	SEP	Turtles/Flotsam	3	9	3	15
Total			24	84	61	169

a Some samples failed during either COI or RAD analyses; therefore, the same set of individuals was not used in both analyses.

b See Figure 1 for specific collection sites within each region and region abbreviations.

c For genetic analyses, only one specimen per host (piece of flotsam or turtle) was analyzed, unless absolutely necessary.

d Old specimens, DNA too degraded for RADseq.

Prior to evaluating genetic patterns within *Planes *and *Pa. laevimanus* using genomic RADseq methods, we conducted a single‐locus mtDNA phylogenetic analysis within the family Grapsidae to (a) evaluate statistical support for the relationship between *Pa. laevimanus *and *Planes *(as in Schubart, 2011; Ip et al., 2015), but with wider geographic and taxonomic sampling, (b) evaluate mtDNA phylogenetic patterns within *Planes/Pa. laevimanus*, and (c) quantify and compare the degree of intraspecific genetic variation in mtDNA between clades/species within *Planes*/*Pa. laevimanus* and other grapsid species. Mitochondrial sequences for *Pa. laevimanus *and *Planes *were generated specifically for this study, with additional mtDNA sequences from 168 specimens representing 19 other grapsid species provided by the Florida Museum of Natural History, University of Florida (see Appendix S1 for details). Genomic DNA extractions and mtDNA amplification and sequencing of these museum specimens followed the same methodologies that were used for *Planes *and *Pa. laevimanus *in this study.

### 2.2 DNA extraction

Genomic DNA was extracted from either leg muscle tissue or whole/partial leg samples using one of three methods: (a) DNAzol (muscle tissue samples; Molecular Research Center, Inc. Cincinnati, OH USA), (b) phenol–chloroform–isoamyl alcohol (whole or partial leg samples), or (c) Zymo Research Genomic DNA Microprep kits (whole or partial leg samples; Zymo Research Corporation, Irvine, CA, USA). For each method, extractions were performed according to the manufacturer's recommendations. For (b) and (c), extractions were performed following powderization in a Geno/Grinder 2010 (SPEX SamplePrep, Metuchen, NJ, USA).

### 2.3 COI amplification, sequencing, and analyses

A 650‐bp barcoding fragment of the mitochondrial *cytochrome c oxidase subunit I *(COI) gene was PCR amplified using degenerate universal metazoan primers (forward/reverse: dgLCO/dgHCO) following protocols described in Evans and Paulay (2012). Samples producing PCR products of appropriate size were Sanger sequenced bidirectionally.

Forward and reverse sequences were assembled using Sequencher v4.10.1 (Gene Codes Corporation Ann Arbor, MI, USA) and manually checked for ambiguous and erroneous base calls. Resulting high‐quality COI sequences for *Pa. laevimanus *(*N* = 6), *Pl. marinus *(*N* = 11), *Pl. minutus *(*N* = 30), and *Pl. major *(*N* = 38) (Table 1) were combined with COI sequences from 168 individuals representing 19 other grapsid species and aligned using MUSCLE (Edgar, 2004). A maximum‐likelihood (ML) phylogenetic analysis was carried out in RAxML v 8.0.0 (Stamatakis, 2014) using the default GTRGAMMA model, 1,000 bootstrap replicates, and the rapid bootstrap and ML tree search algorithm (option ‐f a). Within each clade/species supported with at least 60% bootstrap support, nucleotide diversity (*π*) was estimated in *Arlequin* v 3.5 (Excoffier & Lischer, 2010). A minimum spanning haplotype network was also calculated from the COI alignment of *Pa. laevimanus *and *Planes *sequences using PopART (Leigh & Bryant, 2015).

### 2.4 Creation and sequencing of RAD libraries

Genomic DNA quality was checked on agarose gels to ensure the majority of DNA fragments were of high molecular weight. Samples not meeting this criterion were excluded from RAD library development as degraded DNA has been shown to dramatically reduce the ability to recover comparable loci among individuals (Graham et al., 2015). RAD libraries were prepared following the double‐digest (ddRAD) protocols described by Parchman et al. (2012) and Peterson, Weber, Kay, Fisher, and Hoekstra (2012). For each sample, genomic DNA was double digested using EcoRI and MseI restriction enzymes and custom adaptors with unique variable length inline barcodes (8–10 bp) were ligated to resulting fragments. Following PCR enrichment of the library using iProof High‐Fidelity DNA polymerase (Bio‐Rad Hercules, CA, USA), final library products from each individual (6 µl) were pooled, size selected for a fragment range of 250–450 bp, and sequenced on an Illumina HiSeq 2000 1x100 (Illumina, San Diego, CA, USA).

### 2.5 Processing of sequenced RAD tags

Sequence quality filtering and processing were performed using tools in the FASTX‐Toolkit (Gordon & Hannon, 2010). Sequences were filtered to retain reads with a minimum Phred score of 20 for 90% of the read, demultiplexed with zero barcode mismatches, and trimmed to remove the inline barcode and restriction cut‐sites resulting in 84‐bp reads. Sequence alignment, single nucleotide polymorphism (SNP) discovery, and genotyping were performed in STACKS v. 1.21 (Catchen, Amores, Hohenlohe, Cresko, & Postlethwait, 2011; Catchen, Hohenlohe, Bassham, Amores, & Cresko, 2013). In the *ustacks *module, data from each individual were processed allowing a minimum identical read depth of 2 (‐m 2), a maximum distance for allele detection of two (‐M 2), and invoking the –r and –d options. In the *cstacks* module, a master catalog of all observed loci and allelic variants was compiled allowing an initial nucleotide mismatch of two between loci of different individuals (‐n 2). To improve computational efficiency and reduce the quantity of low frequency loci, the catalog was constructed using a subset of individuals (*n* = 35) representing all species and all populations. In the *populations* module, loci were filtered enforcing a minimum stack depth of three (‐m 3), minor allele frequency of 0.05 (‐a 0.05), and a minimum representation in 70% of individuals (‐r 0.7). Alternate parameter values were tested for each module and those used represent a compromise between dataset size, information content, and percentage of missing data (see Appendix S1 for more details).

Because clustering programs can have difficulty identifying lower substructure in the presence of more dominant higher‐level organization (Kalinowski, 2011), different RAD datasets were generated to ensure the retention of loci relevant at both inter‐ and intraspecific scales. Results from the most inclusive analysis were used to inform grouping of individuals for subsequent analyses, making no a priori assumption about species or population designation. The first dataset included all individuals regardless of putative species or ocean region of sampling (RAD dataset 1). Based on the results of this all‐inclusive dataset and the detection of putative hybrid individuals, we then generated two less inclusive datasets to test for additional fine‐scale or hierarchical genetic clustering: RAD dataset 2 (all nonhybrid *Pa. laevimanus *and *Pl. marinus*) and RAD dataset 3 (all nonhybrid *Pl. minutus *and *Pl. major*). While each of the three RAD datasets was assembled using the same parameter settings in each module of STACKS (*ustacks*: –M 2 –m 2; *cstacks*: –N 2 –n 2; and *populations*: ‐m 2 –a 0.05 –r 0.7), the loci retained within each dataset were allowed to change to optimize analyses of population structuring at their respective scale (inter‐ vs. intraspecific scales).

### 2.6 Individual and population clustering

The number of species and/or population clusters present in each dataset was inferred using parametric and nonparametric clustering methods as implemented in the programs STRUCTURE v. 2.3.4 (Pritchard, Stephens, & Donnelly, 2000) and AWCLUST v. 3.0 (Gao & Starmer, 2008), respectively. Both programs provide a means of evaluating different values for *K*, the number of putative genetic clusters (often interpreted as species or populations), but AWCLUST is robust to small sample sizes within putative populations (<10 individuals) and violations of demographic assumptions of Hardy–Weinberg and linkage equilibrium (Deejai, Assawamakin, Wangkumhang, Poomputsa, & Tongsima, 2010; Gao & Starmer, 2008). STRUCTURE and AWCLUST analyses were first performed on the all‐inclusive dataset comprising all putative species and ocean regions (RAD dataset 1), and then separately on the less inclusive datasets (RAD datasets 2 and 3) generated from the results of the first analysis.

In STRUCTURE, 40,000 MCMC generations were run with a burn‐in of 10,000 using an admixture model with correlated allele frequencies, no prior information on sampling location, with five replicates for each value of *K*. STRUCTURE results were processed using STRUCTURE HARVESTER v. 0.06.94 (Earl & vonHoldt, 2012), where different values for *K* were evaluated by comparing the log‐likelihood probability (L(K); mean ± standard deviation) of each model and applying the deltaK method (Evanno, Regnaut, & Goudet, 2005). Based on estimated ancestry coefficients calculated in STRUCTURE, each individual was assigned to one putative species or population cluster at each value of *K*.

In AWCLUST, pairwise allele sharing distance matrices were generated between all individuals in each dataset and multidimensional scaling plots were constructed to visualize putative clusters and identify outliers. Gap statistics were calculated and compared for each value of *K* following 100 null simulations, and each individual was assigned to one putative species or population cluster based on hierarchical clustering plots. In both STRUCTURE and AWCLUST, we tested values of *K* between 1 and 10 for RAD dataset 1, between 1 and 5 for RAD dataset 2, and between 1 and 8 for RAD datasets 3. At each value of *K*, we compared the composition of individuals within clusters to quantify the congruence between STRUCTURE and AWCLUST assignments and to identify common and erroneous clusters based on putative species designations and geography. To test for additional hierarchical structure in each RAD dataset, we also ran ML phylogenetic analyses on the SNP multiple sequence alignments in RAxML v 8.2.10 (Stamatakis, 2014) using an ascertainment bias‐corrected GTRGAMMA model with the Felsenstein correction and the rapid bootstrap algorithm with 300 bootstrap iterations (see Appendix S1 for more details).

### 2.7 Population genomic analyses

For each RAD dataset, the genetic diversity within clusters and genetic differentiation between clusters detected in STRUCTURE and AWCLUST were estimated by calculating pairwise genetic distance (*F*_ST_), observed and expected heterozygosity, and number of private alleles in the program *Arlequin*. Significant differences in *F*_ST_ values among clusters were determined by a 1,000 permutation test with Bonferroni corrections for multiple comparisons in *Arlequin*.

For RAD dataset 3, we performed additional fine‐scale analyses based on the observed genetic clustering. A hierarchical analysis of molecular variance (AMOVA; Excoffier, Smouse, & Quattro, 1992) was performed to test how genetic variation is partitioned within and among ocean basins and regions. Regional designations for the AMOVA follow Table 1 and Figure 1, except for the southeast and southwest Atlantic (SEA and SWA) and southeast and southwest Indian (SEI and SWI), which were grouped together into South Atlantic (SA) and Indian (IND), respectively, due to small sample sizes. Significant differences in genetic differentiation (*F*_ST_) were evaluated using a Bonferroni correction alpha value of 0.0009. To test for isolation by distance, Mantel tests implemented in GENALEX v. 6.5 (Peakall & Smouse, 2012) were conducted between *F*_ST_ and log‐transformed geographic distance among sites within the North Atlantic and Pacific oceans. Last, we used the SNP multiple sequence alignment for RAD dataset 3 to generate a phylogenetic network with the Neighbor‐Net algorithm in SPLITSTREES v 4.14.6 (Huson & Bryant, 2006).

## 3 RESULTS

### 3.1 COI phylogenetic analysis

**Figure 2:**
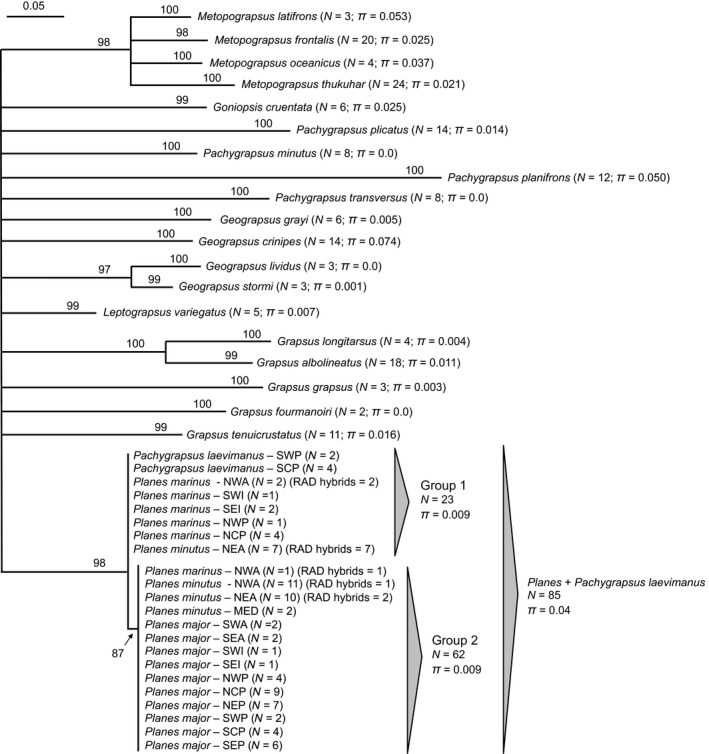
Maximum‐likelihood phylogenetic analysis of the mitochondrial gene COI for the family Grapsidae. Numbers at nodes indicate bootstrap support values and nodes with <60% bootstrap support are collapsed. Numbers at tips indicate sample sizes (*N*) and estimates of nucleotide diversity (*π*) within each clade. Uninformative, short branches within Group 1 and Group 2 are not shown to combine sequences by sampling locations

A ML phylogenetic analysis of 253 COI sequences from 23 grapsid species (including *Pa. laevimanus *and three putative *Planes *species) resulted in consistently high bootstrap support (≥97%) for the monophyly of most species. However, COI data provide little to no resolution of relationships between genera or within species after collapsing nodes with weak bootstrap support (<60%) (Figure 2). In this analysis, *Pa. laevimanus* and *Planes* form a single clade that is distinct from other grapsid species with high bootstrap support (98%), which is consistent with the paraphyly of *Planes *due to the well‐supported inclusion of *Pa. laevimanus *as found by Schubart (2011) and Ip et al. (2015). All COI sequences for *Pa. laevimanus *and *Planes *are available on GenBank (Accessions MH931286‐MH931370).

**Figure 3:**
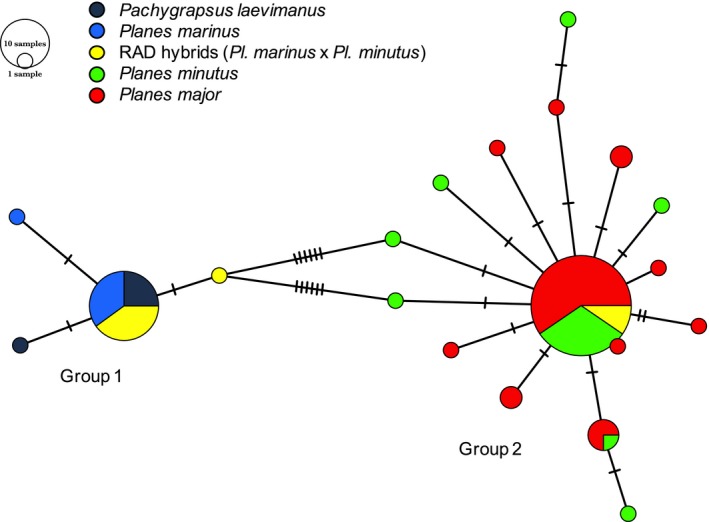
Minimum spanning haplotype network of the mitochondrial gene COI for *Pachygrapsus laevimanus *(*N* = 6) and *Planes* (*N* = 79). Different colors indicate putative species designations or “RAD hybrids” identified in subsequent genomic analyses, and bars indicate haplotype differences

Within the clade that unites *Planes *and *Pa. laevimanus*, we found one strongly supported polytomy (bootstrap = 87%) nested within a larger polytomy (Figure 2). Individuals within the nested clade (Group 2 in Figure 2) were united because they shared seven unique mtDNA SNPs that were not found in individuals outside the nested clade (Group 1 in Figure 2). Group 1 comprised mostly individuals of *Pa. laevimanus* (*N* = 6) and *Pl. marinus *(*N* = 10), but also seven *Pl. minutus *individuals from the Northeast Atlantic (NEA). Group 2 comprised mostly individuals of *Pl. minutus *(*N* = 23) and *Pl. major *(*N* = 38), but also one *Pl. marinus *individual from the Northwest Atlantic (NWA). The nucleotide diversity within each of these groups (*π* = 0.009) and within the entire *Planes/Pa. laevimanus *clade (*π* = 0.044) was similar to or less than that of other grapsid species (*π* = 0.0–0.074) at this mtDNA locus (Figure 2). There was no support for substructuring based on putative species designations or geography within either group inside the *Planes/Pa. laevimanus *clade. Results from the COI haplotype network support the patterns found in the ML phylogenetic analysis: consistent differences between individuals in Group 1 and Group 2 with some minor, uninformative variation at the individual level within each group (Figure 3).

Subsequent RADseq analyses revealed the presence of genetic hybrids. These individuals, identified in Figures 2 and 3 as “RAD hybrids,” showed significant genomic admixture (ancestry coefficients = 5%–95%) between one RADseq cluster comprised of mostly *Pa. laevimanus/Pl. marinus *and another comprised of mostly *Pl. minutus/Pl. major*. Within mtDNA Group 1, all *Pl. marinus *from the NWA (*N* = 2) and *Pl. minutus *from the NEA (*N* = 7) were identified as hybrids. Within Group 2, the single *Pl. marinus *from the NWA, a *Pl. minutus *in the NWA (one out of 11 individuals) and NEA (two out of 10 individuals) were identified as hybrids. An additional two hybrids from NEA were not sequenced for COI. Therefore, all individuals (*N* = 8) for which the morphological designation did not match mtDNA group were identified as hybrids. Excluding hybrids from the COI phylogeny (Figure 2) and haplotype network (Figure 3), Group 1 comprised only *Pa. laevimanus *and *Pl. marinus*, and Group 2 comprised only *Pl. minutus *and *Pl. major. *See Appendix S2 for more information on morphology.

### 3.2 RAD libraries and processing

We sequenced RAD libraries for 152 individuals in two lanes of Illumina HiSeq 2000, generating 297 million raw reads. After quality filtering, we retained 215 million reads from 145 individuals: 6 *Pa. laevimanus*, 11 *Pl. marinus*, 52 *Pl. minutus,* and 76 *Pl. major* (Table 1). The mean number of filtered reads per individual was 903,186 with STACKS utilizing on average 718,070 for loci discovery and allele calling, resulting in an average of 114,409 loci per individual with an average read depth of 6.0 (Table S1). All RADseq reads were accessioned in the short read archive in GenBank under BioProject No. PRJNA471559 (Accessions SAMN09211878‐SAMN09212022; data matrices can be made available upon request).

We assembled RAD datasets de novo first including all putative species and ocean regions (RAD dataset 1; *N* = 145), and then, based on the clustering and genetic analysis of this most inclusive dataset, two additional less inclusive datasets were constructed in order to evaluate further substructure. The putative hybrids were excluded in the two less inclusive datasets, which comprised *Pa. laevimanus *and *Pl. marinus *individuals (RAD dataset 2; *N* = 14) and *Pl. minutus *and *Pl. major *individuals (RAD dataset 3; *N* = 116). For the three RAD datasets, we recovered the following numbers of loci: RAD dataset 1 = 1,108 loci; RAD dataset 2 = 3,314 loci; and RAD dataset 3 = 1,288 loci. Most loci were unique to each dataset, while some loci were shared among datasets (Figure S1). RAD dataset 1 contained a high proportion of loci with elevated haplotype diversity as calculated by Φ_ST_ values (Figure S2), suggesting this dataset contained a larger number of loci with fixed differences appropriate for evaluating higher‐level (i.e., species‐level) relationships. RAD datasets 2 and 3 contained a substantially higher proportion of loci with lower Φ_ST_ values (Figure S2), suggesting that these datasets were more appropriate for assessing recent divergences and finer population‐level patterns.

### 3.3 Clustering of individuals and populations

The optimal values for *K*, the number of putative species or population clusters, in each dataset were not always clear‐cut within and between STRUCTURE and AWCLUST analyses. Therefore, instead of selecting and analyzing just one seemingly optimal value of *K*, thereby excluding other potentially important patterns, we analyzed results at multiple *K* values within each dataset.

**Figure 4:**
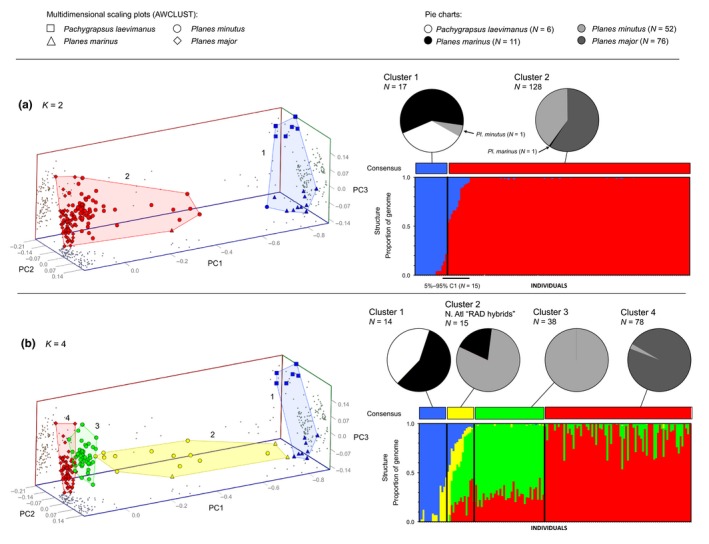
Results from clustering analyses of RAD dataset 1 at (a) *K* = 2 and (b) *K* = 4. Multidimensional scaling plots (from AWCLUST) show individuals distributed along three principal coordinate axes with different colors indicating different putative clusters and different icon shapes indicating different putative species (small gray dots indicate the positions of each point along each pair of axes). STRUCTURE bar plots show the proportion of the genome of each individual (*x*‐axis) that originates from each putative cluster, and black bars separate individuals into different clusters at each value of *K*. Colored bars above STRUCTURE bar plots show the consensus cluster assignment for each individual, and pie charts show the putative species composition of each cluster at each value of *K*. Cluster numbers above pie charts correspond with numbers on scaling plots

For RAD dataset 1, which comprised all putative species and ocean regions, we found support for *K* = 2 in STRUCTURE and *K* = 4 in AWCLUST (Figure S3). At *K* = 2, there was high congruence (≥95%) between STRUCTURE and AWCLUST assignments (i.e., individuals grouped in similar clusters in both analyses) and most individuals segregated into two putative species clusters: One comprised of mostly *Pa. laevimanus/Pl. marinus *and another comprised of mostly *Pl. minutus/Pl. major* (Figure 4a). However, there were 15 individuals—all from either the NEA or NWA—that showed significant admixture between the two putative species clusters (ancestry coefficients = 5%–95%; Figure 4a), suggesting that the genomic composition of these individuals may be the result of hybridization. At *K* = 3 (not shown) and *K* = 4 (Figure 4b), which both showed high congruence (≥95%) between STRUCTURE and AWCLUST assignments, these 15 putative hybrid individuals formed a distinct cluster. Recent hybridization between the two putative species clusters identified in this analysis is corroborated by mtDNA data. Thirteen of the 15 putative “RAD hybrids” are shown in the COI analyses (Figures 2 and 3): Nine carried the *Pa. laevimanus/Pl. marinus* or Group 1 mitochondrial genome, four carried the *Pl. minutus/Pl. major* or Group 2 mitochondrial genome, and two were not sequenced for COI. At *K* = 4 (Figure 4b), the *Pl. minutus/Pl. major* cluster showed further segregation (but with considerable admixture) corresponding primarily to different ocean basins and a priori species designations (see RAD dataset 3 analysis below). Results from the phylogenetic analysis of RAD dataset 1 supported the overall patterns found in the clustering analyses at *K* = 2: two well‐support species groups (*Pa. laevimanus/Pl. marinus *and *Pl. minutus/Pl. major*) with a group of hybrid individuals with varying degrees of similarity between the two species groups (Figure S4; see Appendix S2 for more information).

For RAD dataset 2, which comprised nonhybrid *Pa. laevimanus *(*N* = 6) and *Pl. marinus *(*N* = 8), we found support for *K* = 2 and *K* = 3 in both STRUCTURE and AWCLUST (Figure S3) with 100% congruence between STRUCTURE and AWCLUST assignments. At *K* = 2 (Figure 4a), intertidal *Pa. laevimanus *clearly segregated from rafting *Pl. marinus *with almost no admixture. At *K* = 3 (Figure 4b), *Pa. laevimanus *remained distinct, while *Pl. marinus *segregated geographically into Indian (*N* = 3) and Pacific Ocean (*N* = 5) clusters with some admixture. Results from the phylogenetic analysis of RAD dataset 2 support the overall patterns found in the clustering analyses at *K* = 3 (Figure S5).

For RAD dataset 3, which comprised nonhybrid *Pl. minutus *(*N* = 39) and *Pl. major *(*N* = 77), we found support for *K* = 2 and *K* = 4 in STRUCTURE and AWCLUST (Figure S3) with high congruence (>95%) between STRUCTURE and AWCLUST assignments at all values of *K*. At *K* = 2 (Figure 4a), most individuals segregated into two geographic clusters with some admixture: One comprised mostly of individuals from the North Atlantic (including the Mediterranean Sea) and another comprised mostly of individuals from the Pacific, with no consistent segregation of individuals from the South Atlantic and Indian oceans into either cluster (ancestry coefficients = 40%–60%). At *K* = 4 (Figure 4b), individuals segregated roughly into four geographic clusters: (a) North Atlantic and Mediterranean Sea, (b) South Atlantic and Indian, (c) West Pacific, and (d) East Pacific. There were considerable overlap and admixture between the two Pacific clusters, with individuals from both clusters being found in each of the three regions in the North Pacific (Figure 4b). Additional fine‐scale or hierarchical genetic clustering was tested for within the North Atlantic Ocean (RAD dataset 3, Cluster 1) and Pacific Ocean (RAD dataset 3, Clusters 3 and 4) using STRUCTURE and AWCLUST, but no significant support for any additional substructuring in either analysis was found. See Appendix S2 for additional tests supporting the consistency of this clustering pattern (*K* = 4), as well as the exclusion of erroneous clusters at higher values of *K*. Results from the phylogenetic analysis of RAD dataset 3 showed weakly supported geographic groupings, but no well‐supported nodes (Figure S6), which is characteristic of phylogenetic analyses in which genetic exchange between populations is ongoing or very recent.

### 3.4 Population genomic analyses

For each RAD dataset, all pairwise comparisons of genetic differentiation (*F*_ST_) between clusters identified in STRUCTURE and AWCLUST were highly significant (*p*‐value <0.001). Table 2 shows pairwise comparisons of genetic distance (*F*_ST_), and associated *p*‐values, observed and expected heterozygosity, and number of private alleles among clusters identified in RAD dataset 1 (Figure 4). See additional information in Appendix S2 and results for *K* = 3 in Table S2. Collectively, the results from RAD dataset 1 suggest the presence of two species groups (*Pa. laevimanus/Pl. marinus *and *Pl. minutus*/*Pl. major*; *F*_ST_ =0.727) and a zone of hybridization between the two in the North Atlantic (*F*_ST_ = 0.244 and 0.400), as well as additional weak ocean‐specific differentiation within the *Pl. minutus*/*Pl. major* species group that was also consistent with a priori species designations (*F*_ST_ = 0.099).

**Table 2:** Pairwise comparison of genetic distance (*F*_ST_; below diagonal) and associated *p*‐values (above diagonal), observed and expected heterozygosity (*SE* = standard error), and number of private alleles among clusters identified in RAD dataset 1

	Clusters				*H*_o_ (*SE*)	*H*_e_ (*SE*)	Pr
*K* = 2 (Figure 4a)	1	2					
Cluster 1—*Pa. laevimanus* +* Pl. marinus* * * *+ 3* “RAD hybrids”	–	<0.0001					
Cluster 2—* Pl. minutus* +* Pl. major* + 12 “RAD hybrids”	0.683	–					
*K* = 4 (Figure 4b)	1	2	3	4			
Cluster 1—*Pa. laevimanus *+* Pl. marinus*	–	<0.0001	<0.0001	<0.0001	0.068 (0.021)	0.127 (0.032)	14
Cluster 2—“RAD hybrids”	0.244	–	<0.0001	<0.0001	0.253 (0.041)	0.309 (0.032)	0
Cluster 3—*Pl. minutus*	0.728	0.358	–	<0.0001	0.095 (0.030)	0.102 (0.021)	5
Cluster 4—*Pl. minutus* +* Pl. major*	0.763	0.419	0.099	–	0.086 (0.032)	0.090 (0.021)	14

Note

*H*_o_, Observed heterozygosity; *H*_e_, expected heterozygosity; Pr, number of private alleles.

**Figure 5:**
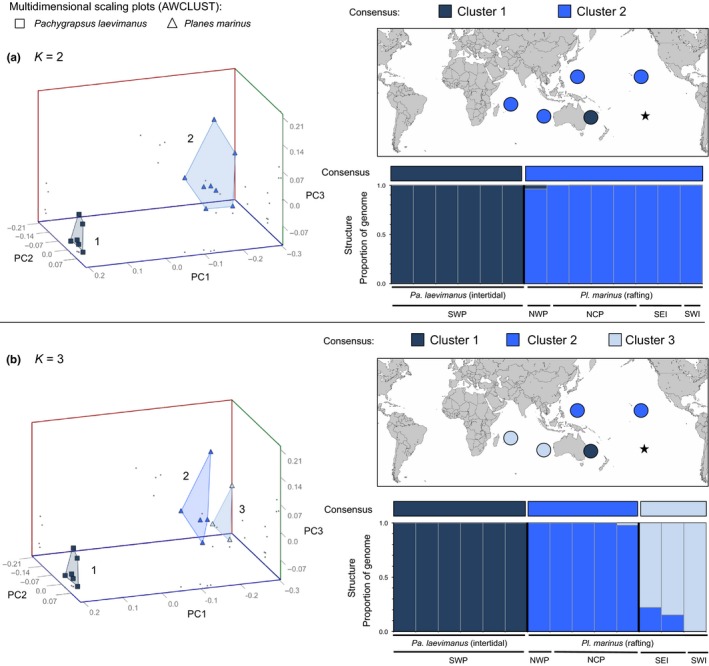
Results from clustering analyses of RAD dataset 2 at (a) *K* = 2 and (b) *K* = 3. Multidimensional scaling plots (from AWCLUST) show individuals distributed along three principal coordinate axes with different colors indicating different putative clusters and different icon shapes indicating different putative species (small gray dots indicate the positions of each point along each pair of axes). STRUCTURE bar plots show the proportion of the genome of each individual (*x*‐axis) that originates from each putative cluster, and black bars separate individuals into different clusters at each value of *K*. Labels below STRUCTURE bar plots show the putative species designation and geographic region of each individual. Colored bars above STRUCTURE bar plots show the consensus cluster assignment for each individual and pie charts on the map show the composition of individuals from different putative clusters in different geographic locations. Cluster numbers above map correspond with numbers on scaling plots. Black star indicates Rapa Island from which *Pachygrapsus laevimanus* specimens were included in this study, but failed during RADseq development

Table 3 shows pairwise comparisons of genetic distance (*F*_ST_), and associated *p*‐values, observed and expected heterozygosity, and number of private alleles among clusters identified in RAD dataset 2 (Figure 5). Collectively, the results from RAD dataset 2 suggest that intertidal *Pa. laevimanus *are distinct from rafting *Pl. marinus *(*F*_ST_ = 0.261), which differentiate further by ocean basin (Pacific vs. Indian; *F*_ST_ = 0.215).

**Table 3:** Pairwise comparison of genetic distance (*F*_ST_; below diagonal) and associated *p*‐values (above diagonal), observed and expected heterozygosity (*SE* = standard error), and number of private alleles among clusters identified in RAD dataset 2

	Clusters			*H*_o_ (*SE*)	*H*_e_ (*SE*)	Pr
*K* = 2 (Figure 4a)	1	2				
Cluster 1—*Pa. laevimanus *(intertidal)	–	<0.0001				
Cluster 2—* Pl. marinus* (rafting)	0.261	–				
*K* = 3 (Figure 4b)	1	2	3			
Cluster 1—*Pa. laevimanus* (intertidal)	–	<0.0001	<0.0001	0.076 (0.003)	0.147 (0.003)	1,196
Cluster 2—* Pl. marinus* (rafting; *N*. Pacific)	0.272	–	<0.0001	0.084 (0.003)	0.133 (0.003)	657
Cluster 3—* Pl. marinus* (rafting; Indian)	0.387	0.215	–	0.113 (0.004)	0.207 (0.004)	750

Note

*H*_o_, observed heterozygosity; *H*_e_, expected heterozygosity; Pr, number of private alleles.

**Figure 6:**
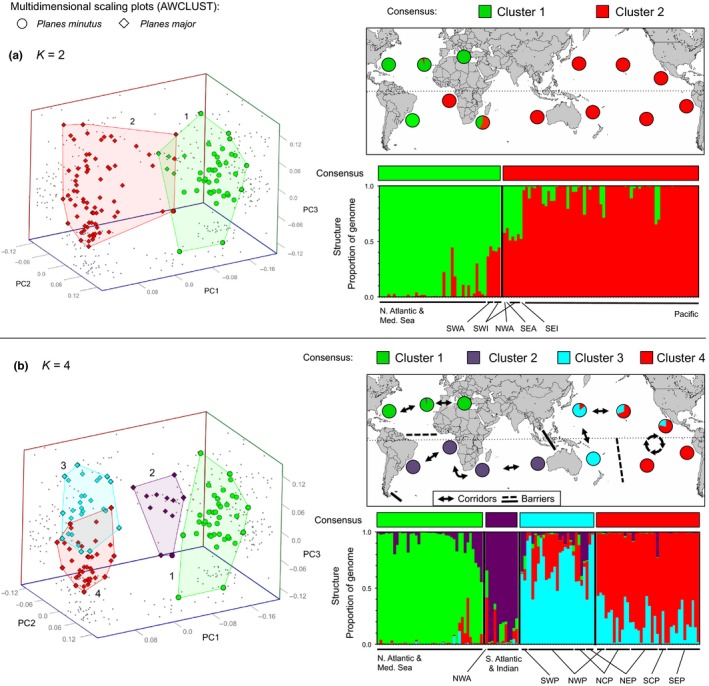
Results from clustering analyses of RAD dataset 3 at (a) *K* = 2 and (b) *K* = 4. Multidimensional scaling plots (from AWCLUST) show individuals distributed along three principal coordinate axes with different colors indicating different putative clusters and different icon shapes indicating different putative species (small gray dots indicate the positions of each point along each pair of axes). STRUCTURE bar plots show the proportion of the genome of each individual (*x*‐axis) that originates from each putative cluster, and black bars separate individuals into different clusters at each value of *K*. Labels below STRUCTURE bar plots show the putative species designation and geographic region of each individual. Colored bars above STRUCTURE bar plots show the consensus cluster assignment for each individual, and pie charts on the map show the composition of individuals from each putative cluster in different geographic locations. Cluster numbers above map correspond with numbers on scaling plots. Arrows indicate dispersal corridors, and bars indicate dispersal barriers (solid > dashed, in terms of genetic discontinuity)

Table 4 shows pairwise comparisons of genetic distance (*F*_ST_), and associated *p*‐values, observed and expected heterozygosity, and number of private alleles among clusters identified in RAD dataset 3 (Figure 6). See additional information in Appendix S2 and results for *K* = 3 in Table S3. This analysis from RAD dataset 3 shows an overall pattern of relatively low, but consistent, genetic differentiation between the four ocean regions with evidence of subtle differentiation between *Pl. minutus *(Cluster 1) and *Pl. major *(Clusters 2, 3 and 4; *F*_ST_ = 0.0.086, 0.123 and 0.156, respectively) that was comparable to differentiation within *Pl. major *(Cluster 2 vs. 3 and 4; *F*_ST_ = 0.080 and 0.088). Results from the SNP‐based Neighbor‐Net support the overall patterns found in the clustering analyses at *K* = 4 (Figure S7), but with a highly complex network along the backbone that suggests considerable ongoing gene flow between clusters.

**Table 4:** Pairwise comparison of genetic distance (*F*_ST_; below diagonal) and associated *p*‐values (above diagonal), observed and expected heterozygosity (*SE* = standard error), and number of private alleles among clusters identified in RAD dataset 3

	Clusters				*H*_o_ (*SE*)	*H*_e_ (*SE*)	Pr
*K* = 2 (Figure 4a)	1	2					
Cluster 1—*Pl. minutus* (NWA, NEA, MED) + *Pl. major* (SWA, SWI)	–	<0.0001					
Cluster 2—* Pl. minutus* (NWA) + *Pl. major* (SEA, SWI, SEI, Pacific)	0.122	–					
*K* = 4 (Figure 4b)	1	2	3	4			
Cluster 1—*Pl. minutus* (NWA, NEA, MED)	–	<0.0001	<0.0001	<0.0001	0.188 (0.006)	0.213 (0.004)	29
Cluster 2—*Pl. minutus* (NWA) + *Pl. major* (SWA, SEA, SWI, SEI)	0.086	–	<0.0001	<0.0001	0.179 (0.006)	0.197 (0.005)	2
Cluster 3—*Pl. major* (NWP, SWP, NCP, NEP)	0.123	0.080	–	<0.0001	0.191 (0.006)	0.207 (0.004)	0
Cluster 4—*Pl. major* (NWP, NCP, SCP, NEP, SEP)	0.156	0.088	0.038	–	0.174 (0.006)	0.198 (0.004)	5

Note

H_o_, observed heterozygosity; H_e_, expected heterozygosity; Pr, number of private alleles.

**Figure 7:**
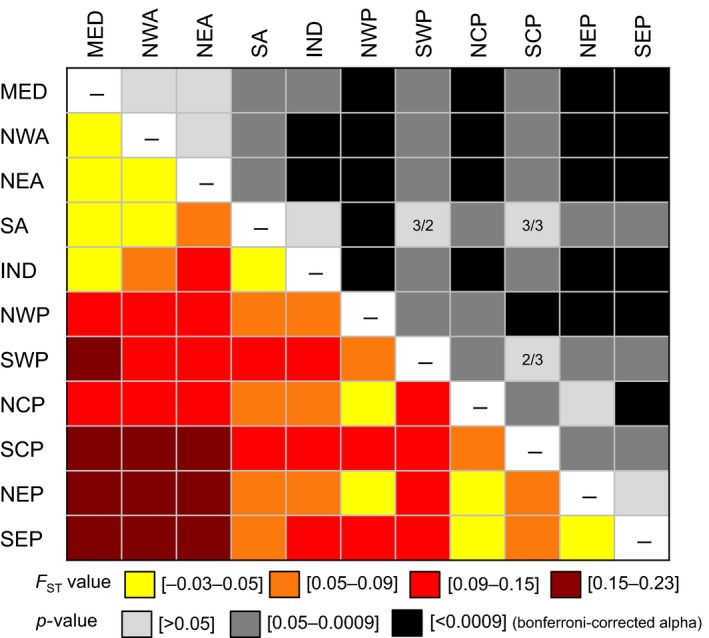
Heatmap showing pairwise comparisons of genetic distance (*F*_ST_; below diagonal) and associated *p*‐values (above diagonal) for 11 ocean regions from AMOVA. SEI and SWI regions (Figure 1) are combined into IND (Indian Ocean), and SWA and SEA were combined into SA (South Atlantic). *F*_ST_ values correspond with significant differences at a Bonferroni‐corrected alpha value of 0.0009, except when both samples sizes are small (see numbers in cells; row/column). See Table S4 for exact values

Additionally, for RAD dataset 3, an analysis of molecular variance (AMOVA) across 11 regions in three ocean basins showed that the majority of genetic variation was found among individuals within regions (87%) and that there was considerably more genetic variation between oceans (11%) than among regions within oceans (2%) (Table 5). Patterns of genetic differentiation (*F*_ST_) among the regions designated in the AMOVA (Figure 7; Table S4) were generally consistent with patterns (and associated *F*_ST_ values) among clusters identified in STRUCTURE and AWCLUST (Figure 6; Table 4). We also found no correlation between genetic and geographic distance within either the North Atlantic Ocean (Mantel test: *y* = −0.0112*x* + 0.03; *r* ^2^ = 0.334; *p*‐value = 0.17) or Pacific Ocean (Mantel test: *y* = 0.014*x* − 0.04; *r* ^2^ = 0.008; *p*‐value = 0.21). Collectively, the results of RAD dataset 3 suggest that *Pl. minutus *and *Pl. major* are a single, globally distributed species that shows some geographic structure with weak genetic differentiation among widely separated aggregations.

**Table 5:** Analysis of molecular variance (AMOVA) among 11 regions. Two pairs of oceanic regions were combined due to sample sizes (SEA and SWA in South Atlantic; SEI and SWI into Indian)

Sources of variation	Degrees of freedom	Variance	Percentage of variation	*p*‐Value
Between oceans	3	9.54	11.11	0.0019
Among regions within oceans	7	1.58	1.83	<0.0001
Among individuals within regions	221	74.80	87.06	<0.0001
Total	231	85.92	–	–

## 4 DISCUSSION

A major challenge in phylogeography is predicting the role that dispersal mode plays in shaping population structure and ultimately species diversification (Palumbi, 1994, 2003 ). In this study, we use rafting crabs to test whether the ability to disperse as pelagic larvae and as adults associated with oceanic flotsam and sea turtles facilitates transoceanic genetic exchange, thereby limiting both species diversity within this group of crabs and intraspecific genetic differentiation among widely separated populations. Because initial mtDNA analyses showed only limited divergence between species and no evidence of population structure, it was impossible to distinguish whether this was caused by ongoing genetic exchange or because the mtDNA marker simply lacked the resolution to detect existing phylogeographic patterns. We addressed this problem using genomewide SNP data. We found convincing evidence that (a) intertidal *Pa. laevimanus* is sister to rafting *Pl. marinus*, (b) *Pl. minutus *and *Pl. major *comprise a single globally distributed species that is sister to *Pa. laevimanus *and *Pl. marinus*, and that hybridizes with *Pl. marinus *in the North Atlantic, and (c) *Pl. minutus/major *exhibit limited population structure at a global scale with weak genetic discontinuities associated with prominent oceanographic features. Our results show how life history changes that augment dispersal potential (i.e., adults shifting from intertidal to rafting) can limit, but not prevent, species diversification and population differentiation, and highlight the value of genomic data in resolving phylogeographic patterns in organisms with large, highly connected populations.

### 4.1 Low species diversity in rafting crabs

Our results confirm that the genus *Planes *is paraphyletic due to its well‐supported relationship with *Pa. laevimanus* (Ip et al., 2015; Schubart, 2011), only with greater taxonomic and geographic depth. The morphological similarity between *Planes*, especially *Pl. marinus*, and *Pachygrapsus *has led to taxonomic confusion in the past (Chace, 1951, 1966 ). However, *Pa. laevimanus *has never been linked to *Planes *until genetic data were analyzed (Schubart, 2011; Ip et al., 2015; this study). In a novel observation, the affinity between *Pa. laevimanus *and *Planes *is evident when comparing male gonopod morphologies: *Pa. laevimanus *is clearly more similar in shape to *Planes *(Figure 2 in Chace, 1951) than to any *Pachygrapsus* (Figure 15 in Poupin et al., 2005). Like many groups of marine animals, the use of external morphology and traditional genotypic markers has failed to produce a resolved phylogenetic hypothesis for the family Grapsidae (e.g., Schubart, Cuesta, & Felder, 2002; Schubart, Cannicci, Vannini, & Fratini, 2006; Schubart, 2011; Ip et al., 2015; this study). The application of phylogenetic RADseq (e.g., Jones, Fan, Franchini, Schartl, & Meyer, 2013; Wagner et al., 2013) in conjunction with classical morphological analyses will help resolve phylogenetic patterns, thereby providing the framework to investigate pressing evolutionary questions within this family and in other taxonomically challenging groups.

Our RADseq analyses indicate that intertidal *Pa. laevimanus *is sister to rafting *Pl. marinus*. In the absence of any differences in the mitochondrial genome or at least at the COI locus—a fast‐evolving locus often used as a species‐level barcode (Evans & Paulay, 2012)—our interpretation of these results is that there has been a recent and rapid speciation event, and likely concomitant selection for traits associated with different habitats: wider carapace and the lack of natatory fringes in *Pa. laevimanus *(similar to other intertidal *Pachygrapsus *species) and rounder carapace and natatory fringes in *Pl. marinus *(similar to other *Planes *species). However, this current dataset is small and lacks geographic overlap between the two species—no *Pl. marinus* specimens from the southwest Pacific were included. The possibility that *Pa. laevimanus *and *Pl. marinus *are habitat‐specific variants of the same species cannot be ruled out. A more complete sampling regime would allow us to generate more resolved patterns and identify specific adaptive changes in larval recruitment, morphology, and reproductive and social behavior associated with changes in adult habitat and ultimately speciation.

Our mtDNA and RADseq analyses did not support the species‐level distinction between *Pl. minutus *and *Pl. major*. The highly subtle and overlapping morphological traits that were used to separate *Pl. minutus *(only North Atlantic) and *Pl. major* (worldwide, except North Atlantic) may simply be related to geographic variation in body size and concomitant allometric changes in traits related to limb length (as in Chace, 1951), or regional variation or phenotypic plasticity in traits related to masticatory structures (as in Frick, Kopitsky, Bolten, Bjorndal, & Martins, 2011). Alternatively, morphological differences detected in the North Atlantic might have resulted from the inclusion of hybrid individuals during morphological comparisons. Many of the traits that were thought to differentiate *Planes *species tend to place *Pl. minutus* intermediate between *Pl. marinus *and *Pl. major*, suggesting that hybridization between *Pl. marinus *and *Pl. minutus/major *in the North Atlantic leads to subtle morphological differences that have confused morphological taxonomy. The occurrence of hybridization only in one region—the North Atlantic—invokes questions regarding reproductive isolating mechanisms across the rest of the sympatric range of *Pl. marinus *and *Pl. minutus/Pl. major*, which includes all other temperate and tropical oceans of the world. Of particular interest are those instances where *Pl. marinus *and *Pl. minutus /major* share the same raft (Pfaller & Gil, 2016) or the same sea turtle (Frick et al., 2011) yet do not interbreed. Identifying the factors that both promote hybridization in specific areas and deter hybridization elsewhere would shed light on the mechanisms underlying the maintenance and merger of species diversity on a broader scale (Abbott et al., 2013; Barton, 2001).

Despite plausible theoretical expectations for the effect that high dispersal potential should have on diversification (Avise, 2000; Palumbi, 1994, 2003 ), patterns of diversification among planktonic and neustonic organisms are quite variable. Our results for *Planes *are consistent with theoretical predictions, in which the combination of long‐distance dispersal by pelagic larvae and potentially worldwide dispersal of rafting adults and juveniles appears to have limited diversification within the group—only three species, one intertidal and two rafting. Other surface‐ and subsurface‐dwelling oceanic animals show patterns that are both consistent and contradictory to theoretical expectations. Two independent lineages of sea skaters (genus *Halobates*; Insecta) have subsequently speciated following their colonization of the open ocean, although species diversity has remained fairly low (Anderson, Cheng, Damgaard, & Sperling, 2000; Damgaard, Andersen, Cheng, & Sperling, 2000). The amphipod *Caprella andreae*, which like *Planes *is an obligate associate of surface‐drifting oceanic flotsam and sea turtles, shows high diversity and cryptic speciation across a relatively small geographic area compared to *Planes* (Cabezas, Navarro‐Barranco, Ros, & Guerra‐García, 2012). Oceanic nudibranchs display different diversification patterns between sister species: *Glaucus atlanticus *is cosmopolitan and shows no evidence for cryptic diversification, while *Glaucus marginatus *is restricted to the Indo‐Pacific and has diversified into four distinct lineages (Churchill, Alejandrino, Valdes, Foighil, & D., 2013). Lastly, cosmopolitan oceanic copepods (e.g., *Pleuromamma abdominalis *and *Oithona similis*) tend to show extensive cryptic diversity and high rates of endemism (Cornils, Wend‐Heckmann, & Held, 2017; Goetze, 2003; Hirai, Tsuda, & Goetze, 2015).

While examples of diversification patterns among planktonic and neustonic animals are relatively few compared to neritic taxa, there appears to be no ubiquitous pattern for their diversification and only some patterns are consistent with theoretical expectations based on dispersal potential. It is clear that while the capacity for long‐distance dispersal likely plays an important role in limiting opportunities for local adaptation and diversification (as well as extinction), the mechanisms leading to speciation in the open ocean are far more complex and might also involve behavioral changes associated with selection of different temperature and salinity profiles in the pelagic environment (Knowlton, 2000; Palumbi, 1994, 2003 ).

### 4.2 Weak global population structure in rafting crabs

Global patterns of population structure in *Planes *are primarily at the level of major ocean basins, and genetic indices indicate recent and/or ongoing gene flow throughout the temperate and tropical oceans of the world. At this global scale, such weak differentiation indicates that *Planes *populations behave similarly to ubiquitous microbial populations, in which continuous large‐scale dispersal sustains their global distribution and limits biogeographic structure (Finlay, 2002). Conversely, intertidal grapsid crabs that rely exclusively on multistaged pelagic larvae for long‐distance dispersal tend to show little to no transoceanic connectivity (Cassone & Boulding, 2006; Schubart et al., 2005), indicating that large distances across ocean gyres represent significant barriers to pelagic larval dispersal. While *Planes *populations do show subtle genetic discontinuities associated with prominent oceanographic features, our results support the prediction that the ability of adults to disperse while rafting on oceanic flotsam and sea turtles augments pelagic larval dispersal and facilitates transoceanic, if not near global, connectivity.

Subtle genetic discontinuities among globally distributed aggregations of *Planes *highlight potential barriers to rafting dispersal. Differentiation between individuals in the Indian Ocean from those in the Pacific Ocean indicates that the Indonesian Archipelago represents a weak dispersal barrier. The absence of major ocean currents passing through the archipelago and the presence of hundreds of islands likely limits the frequency and success of dispersal across this boundary. The Indonesian Archipelago appears to be a strong dispersal barrier structuring populations of *Halobates micans* (Anderson et al., 2000), but does not result in any detectable genetic differentiation in populations of *Glaucus atlanticus *(Churchill, Valdés, Foighil, & D., 2014). Gyre boundaries may also have a tendency to deflect flotsam back into their respective gyres, therefore reducing the frequency of dispersal by *Planes *between major ocean gyres. However, in the absence of prominent physical barriers, evidence for genetic discontinuity within a species becomes difficult to explain (Lowe & Allendorf, 2010).

The nonpolar distribution of *Planes* likely reflects its inability to survive cold temperatures (Chace, 1951; Spivak & Bas, 1999), thereby limiting dispersal across regions below their thermal minimum (e.g., the Arctic and Southern oceans). Our results show clear, albeit weak, differentiation between individuals in the Atlantic and Pacific oceans, indicating that continental landmasses and the polar waters at Cape Horn (southern South America) limit dispersal. Dispersal limitations across this barrier have led to Atlantic‐Pacific speciation in a tropical rafting crab, *Plagusia *(Schubart, González‐Gordillo, Reyns, Liu, & Cuesta, 2001). However, our results indicate that the cold, but not polar, waters around Cape of Good Hope (southern Africa) do not limit dispersal in *Planes *and instead act as a dispersal corridor. These patterns are only partially consistent with genetic patterns of other neustonic organisms: *Glaucus *nudibranchs and *Halobates *sea skaters show restricted dispersal across both Cape Horn (Atlantic‐Pacific disjunction) and the Cape of Good Hope (Atlantic–Indian disjunction) (Anderson et al., 2000; Churchill et al., 2014). *Planes *may simply have a lower thermal tolerance, thereby allowing dispersal between the Atlantic and Indian oceans. However, *Planes *may also be able to successfully navigate this potential barrier while associated with sea turtles. Both loggerhead and green turtles show genetic connectivity across this biogeographic boundary (Bourjea et al., 2006; Shamblin et al., 2014), providing a potential dispersal vector for *Planes *that is unavailable to other neustonic animals that are not known to associate with sea turtles.

Patterns of genetic structure revealed in our genomic RADseq analyses were not detected in our mtDNA analysis. Traditional genotypic markers (e.g., COI) can provide valuable phylogeographic inferences when there is sufficient genetic resolution to elucidate population‐level differences, which is often the case for organisms that have low connectivity among populations. Because these markers were the primary tool available in the past and estimating population structure is more clear‐cut when populations are distinct, the literature is somewhat biased toward positive examples (Benestan et al., 2015; Goetze, 2005; McCormack et al., 2013). However, when little to no population structure is detected with traditional markers*, *as in *Planes *and other neustonic and planktonic animals [copepods (Bucklin & Kocher, 1996; Bucklin, LaJeunesse, Curry, Wallinga, & Garrison, 1996; Bucklin et al., 2000), euphausiids (e.g., Zane et al., 1998; Zane & Patarnello, 2000; Jarman, Elliott, & McMinn, 2002), squid (Sands, Jarman, & Jackson, 2003), and nudibranchs (Churchill et al., 2014)], it becomes exceedingly difficult to distinguish whether there is ongoing genetic exchange (i.e., panmixis) or whether the marker simply lacks the resolution to detect subtle phylogeographic patterns. For these reasons, our current understanding of the population biology of many neustonic animals remains either unresolved or incomplete. Our results demonstrate the ability of genomic tools, like RADseq, to identify weak population structure when traditional genotypic markers hold no resolution (Fraser 2018). Because these tools are more sensitive to subtleties in phylogeographic structure, they hold great value and future promise for elucidating population‐level patterns in organisms that exhibit vast dispersal potential and high connectivity among distant populations.

## CONFLICT OF INTEREST

None declared.

## AUTHOR CONTRIBUTIONS

JBP, ACP, ABB, and KAB designed the study. JBP, ACP, and SFM performed the research. All authors wrote the paper.

## Data Availability

Genetic data are available on GenBank (COI sequences, Accessions MH931286‐MH931370; RADseq reads, BioProject No. PRJNA471559, Accessions SAMN09211878‐SAMN09212022), and data matrices can be made available upon request (as stated in article). Specimens are available at the Florida Museum of Natural History, University of Florida (Accession # pending; please contact FLMNH for further information) or, if borrowed, at their respective home museums.
