# Supplementary material for: Hitchhiking the high seas: Global genomics of rafting crabs
Source: Ecol Evol. 2019 Jan 23;9(3):957–74. doi: 10.1002/ece3.4694 (PMC6374717; doi:10.1002/ece3.4694)
Supplement: Supplementary file 1 [file ECE3-9-957-s001.docx]

**Appendix for:**

**Hitchhiking the High Seas: Global Genomics of Oceanic Crabs**

Joseph B. Pfaller, Adam C. Payton, Karen A. Bjorndal, Alan B. Bolten and Stuart F McDaniel

**Table of Contents:**

| **Content** | Pages |
| --- | --- |
| **Methods (Appendix S1)** | 2-4 |
| **Results (Appendix S2)** | 5-6 |
| **Tables (S1-4)** | 7-10 |
| **Figures (S1-7)** | 11-17 |

**Methods (Appendix S1)**

**Morphology, Taxon Sampling and Justification**

Each specimen was given an a priori species designation based on external morphology, habitat and/or geography following Chace (1951) and Poupin et al. (2005): (1) *Pa. laevimanus* and *Pl. marinus* were separated by habitat (intertidal versus rafting, respectively), relative carapace length:width (1:>1.2 versus 1:<1.2, respectively) and setation patterns on the walking limbs (no natatory fringe versus natatory fringe, respectively), (2) *Pl. marinus* and *Pl. minutus/Pl. major* were separated by carapace shape and extent of carapace striations (quadrate and distinctly striated versus round and not distinctly striated, respectively) and relative carapace length:width (1:>1.1 versus 1:<1.1, respectively), and (3) *Pl. minutus* and *Pl. major* were separated by geography (North Atlantic and Mediterranean Sea versus South Atlantic, Indian and Pacific, respectively).

Most specimens were collected specifically for this study from 2010 to 2013; however, specimens from unsampled regions or species were also acquired from earlier collections, some of which were initially collected as early as 1995. Specimens were preserved and stored in 70-95% ethanol prior to DNA extraction. Because the duration of storage (0.5-18 yrs) and therefore the extent of DNA degradation were highly variable among specimens, some DNA samples failed during either mitochondrial or genomic analyses. Consequently, the same set of individuals was not used in both analyses (Table 1).

Mitochondrial DNA sequence data from 168 specimens representing 19 other grapsid species were provided by the Florida Museum of Natural History (FLMNH), University of Florida. These included *Geograpsus crinipes* (N = 14), *Geograpsus grayi* (N = 6), *Geograpsus lividus* (N = 3), *Geograpsus stormi* (N = 3), *Goniopsis cruentata* (N = 6), *Grapsus albolineatus* (N = 18), *Grapsus fourmanoiri* (N = 2), *Grapsus grapsus* (N = 3), *Grapsus longitarsus* (N = 4), *Grapsus tenuicrustatus* (N = 11), *Leptograpsus variegatus* (N = 5), *Metopograpsus frontalis* (N = 20), *Metopograpsus latifrons* (N = 3), *Metopograpsus oceanicus* (N = 4), *Metopograpsus thukuhar* (N = 24), *Pachygrapsus minutus* (N = 8), *Pachygrapsus planifrons* (N = 12), *Pachygrapsus plicatus* (N = 14), and *Pachygrapsus transversus* (N = 8). Locality data for each specimen is catalogued by the FLMNH. However, these data are being used for a different study and therefore were not included.

COI Amplification, Sequencing and Analyses

Individual 25 µL reactions (including a negative control) contained 10x buffer, 10mM dNTP, 50 mM MgCl_2_, 10 µM of each primer, 5 units/µL NEB OneTaq Hot Start DNA polymerase (New England Biolabs, Ipswich, MA USA), 1 µL DNA, and a sufficient volume of PCR grade H_2_O to yield a final volume of 25 µL. PCR products were checked on a 1.4% agarose gel to verify the amplification of fragments of appropriate size (500-700 bp). Samples that did not amplify or contained fragments of incorrect size were either re-amplified or excluded from COI sequencing and analyses.

Creation and Sequencing of RAD Libraries

Genomic DNA was digested with two restriction enzymes, EcoRI and MseI (New England Biolabs, Ipswich, MA USA) for 6 hours at 37°C. Individual double-stranded sequence adaptors with unique inline barcodes (8-10 bp with 3bp difference between any two barcodes) were ligated to the 5’ EcoRI overhang of the digested DNA and a universal 3’ adaptor was ligated to the MseI overhang with T4 DNA ligase (New England Biolabs, Ipswich, MA USA) for 6 hours at 16°C. PCR was used to incorporate Illumina flowcell binding sequences and sequencing priming sites to the adaptor ligated DNA fragments using iProof High-Fidelity DNA polymerase (Bio-Rad Hercules, CA USA) with 55°C annealing temperatures and 20 cycles. Barcoded PCR products from each individual (6 µL) were pooled, then gel size selected for fragment ranging from 250-450 bp and sequenced on an Illumina HiSeq 2000 1x100 (Illumina, San Diego, CA USA). Size selection and sequencing were performed by the Interdisciplinary Center for Biotechnology Research at the University of Florida.

Processing of Sequenced RAD Tags

All data processing and analyses were performed on HiPerGator, the University of Florida’s High Performance Research Computing Cluster. Alternate parameter values were tested for each module and those used represent a compromise between dataset size and information content. Increasing minimum read depth (- m) decreased the number of loci found within an individual and increasing nucleotide mismatches at the catalog stage (-n) resulted in increases in the number of polymorphic loci, which increased the number of loci with biologically improbable numbers of alleles (e.g., >5 SNPS or >4 alleles), suggesting paralogs were likely being misidentified as orthologs. While each of the three RAD loci datasets was assembled using the same parameter settings in each module of STACKS (*ustacks*: –M 2 –m 2; *cstacks*: –N 2 –n 2; *populations*: -m 2 –a 0.05 –r 0.7), the loci retained within each dataset was allowed to change to optimize analyses of population structuring at their respective scale. The all-inclusive dataset (RAD dataset 1) will contain some loci that have SNPs that are fixed between groups at a large scale thus providing information for inter specific comparisons, but at smaller scales, such as within group/species, those loci will be uninformative as they contain no polymorphism. Similarly, there will be loci that are polymorphic within one group/species that may be absent entirely from other groups/species such that when a minimum number of individuals required to retain a locus is enforced that locus may be excluded. These loci provide information for intra specific comparisons but may not be present in enough individuals to be valuable for inter specific comparisons. The evolutionary processes acting at these different scales necessitate the use of different datasets, each tailored to addressing the questions at that scale. RAD loci datasets were exported in various file formats from STACKS for subsequent statistical analyses.

**Phylogenetic Analyses of RAD Tags**

As an alternative to clustering methods, maximum likelihood phylogenetic analyses were carried out on each RAD dataset. Multiple sequence alignments of all bi-allelic variant loci were constructed with heterozygous sites coded as IUPAC ambiguities.  Trees were constructed using RaxML v 8.2.10 (Stamatakis 2014) implementing the rapid bootstrap algorithm (-f a) with 300 bootstrap iterations run with an ascertainment bias corrected GTRGAMMA (-m ASC_GTRGAMMA) model of nucleotide evolution implementing the Felsenstein correction for ascertainment bias (--asc_corr=felsenstein).  The bias correction is necessary to account for the use of SNPs being analyzed in a phylogenetic framework.

**References**

Chace, F. A. (1951). The oceanic crabs of the genera *Planes* and *Pachygrapsus*. *Proceedings of the US National Museum, 101*, 65–103.

Poupin, J., Davie, P. J. F., & Cexus, J.C. (2005). A revision of the genus *Pachygrapsus* Randall, 1840 (Crustacea: Decapoda: Brachyura, Grapsidae), with special reference to the Southwest Pacific species. *Zootaxa, 1015*(1), 1–66. doi.org/10.11646/zootaxa.1015.1.1

Stamatakis, A. (2014). RAxML version 8: a tool for phylogenetic analysis and post-analysis of large phylogenies. *Bioinformatics, 30*(9), 1312–1313. doi.org/10.1093/bioinformatics/btu033

**Results (Appendix S2)**

Morphology

In terms of overall morphology, *Pachygrapsus laevimanus* individuals from both Australia and Rapa Island were clearly different from *Pl. marinus* in having a more quadrate carapace (i.e., not laterally convex) that is distinctly wider than long (length:width, 1:>1.2), walking limbs that lack natatory fringes, and setation that is more random and longer. *Planes marinus* and *Planes minutus/major* were differentiated by subtle differences in carapace dimensions (length:width, 1:>1.1 versus 1:<1.1, respectively), shape (quadrate versus round, respectively) and striations (striated versus not striated, respectively). These differences were more distinctive in the Pacific and Indian oceans (i.e., between *Pl. marinus* and *Pl. major* sensu stricto), but were less distinctive in the North Atlantic. The carapaces of *Planes minutus/major* in the North Atlantic were slightly less convex laterally than elsewhere, and the dimensions and shape approached that of *Pl. marinus.*  However, whether or not the carapace was striated remained distinctive between *Pl. marinus* and *Pl. minutus/Pl. major*. Individuals identified as “RAD hybrids” were morphologically intermediate between *Pl. marinus* and *Pl. minutus/Pl. major* in the North Atlantic: carapace only slightly wider than long (length:width, 1:1.0-1.1) and lightly striated in some individuals.

RAD Libraries and Processing

Summary statistics of restriction-site associated DNA-sequencing (RAD*seq*) data processing are shown in Table S1. Datasets contained different sets of loci because we retained only those loci that were present in at least 70% of the individuals in each dataset. Overlap of loci between the three datasets was compared to determine the number of unique loci that were contributing to the analysis at each scale/species group (Fig. S1). Moreover, we plotted the distributions of haplotype divergence (Φ_ST_) to confirm that the composition of loci in each dataset contained variation at the desired evolutionary scale (Fig. S2). Because the probability of recovering shared loci declines as the divergence between the groups being compared increases, datasets with more slowly evolving/mutating loci (high Φ_ST_ values) capture deeper divergences and datasets with more rapidly evolving/mutating loci (low Φ_ST_ values) capture more recent patterns. Many loci retained in RAD datasets 2 and 3 were not retained in Dataset 1, supporting the utility of creating specific datasets addressing questions at different scales.

Clustering of Individuals and Populations

In RAD dataset 1, we also found support for K=6 in AWCLUST, but there was weak support and high variation in L(K) for K=6 in STRUCTURE (Fig. S3), as well as the formation of an erroneous sixth cluster in both analyses (i.e., the cluster was composed of a small number of seemingly random individuals from many geographic locations).

For RAD dataset 3, we tested the consistency of the data to yield support for the observed biogeographic pattern by arranging the loci from most to least variable by Φ_ST_ values. The top half were binned into 5 groups (each representing 10% of the total dataset) and the data were reanalyzed in STRUCTURE (90-100% mean Φ_ST_ = 0.39, 80-90% mean Φ_ST_ = 0.19, 70-80% mean Φ_ST_ = 0.12, 60-70% mean Φ_ST_ = 0.08, 50-60% mean Φ_ST_ = 0.05). This allowed us to evaluate the proportion of loci that were supporting/driving the observed patterns. Regardless of the subset used we consistently found support for K=4 and the aforementioned biogeographic clusters suggesting this pattern is not being driven by a minority of loci with strong differentiation but by the majority of loci spanning a range of variability. Moreover, we tested for additional fine-scale or hierarchical genetic clustering within the North Atlantic Ocean (RAD dataset 3, Cluster 1) and Pacific Ocean (RAD dataset 3, Clusters 3 and 4) using STRUCTURE and AWCLUST, but found no significant support for any additional sub-structuring in either analysis.

**Phylogenetic Analyses of RAD Tags**

In RAD datasets 1 and 3, >85% of the loci were bi-allelic and >97% of the bi-allelic loci passed RAxML filters for variability once recoded to account for heterozygous loci. However, in RAD dataset 2, only 35% of the loci were bi-allelic and 50% of the bi-allelic loci passed RAxML filters for variability, leaving 600 of the original 3314 loci in this phylogenetic analysis.

In RAD dataset 1 (Fig. S4), 39 *Pl. minutus*, 77 *Pl. major* and five RAD hybrids form a clade with high support (bootstrap = 94). Additionally, five *Pl. marinus* from the North Pacific and six *Pa. laevimanus* form a distinct clade (bootstrap = 100) with all *Pa. laevimanus* nested together within this clade (bootstrap = 85). The remaining 10 RAD hybrids, as well as three *Pl. marinus* from the Indian Ocean, lie in a continuum of progressive similarity between the *Pa. laevimanus/Pl. marinus* and *Pl. minutus/Pl. major* clades. We feel that it is most parsimonious to assume that the Indian Ocean *Pl. marinus* may indeed contain loci from a past hybridization event that was insufficient to distinguish them as hybrids at K=2 (ancestry coefficients = 5-95%). These loci, likely not present in RAD dataset 2, differentiate Indian Ocean *Pl. marinus* from Pacific Ocean *Pl. marinus* and *Pa. laevimanus* in RAD dataset 1, but not in RAD dataset 2.

In RAD dataset (Fig. S5) 2, three clades were highly supported: (1) all *Pa. laevimanus* (bootstrap = 100), (2) all *Pl. marinus* (bootstrap = 100), and (3) all *Pl. marinus* from the Indian Ocean (bootstrap = 100). This supports the patterns found in the clustering analyses of RAD dataset 2.

In RAD dataset 3 (Fig. S6), there were no highly supported nodes linking individuals from broad geographic regions. This is indicative of ongoing or recent gene flow among wide-separated geographic regions and is characteristic of intraspecific phylogenetic analyses where population-level differences are weak.

Population Genomic Analyses

In RAD dataset 1 at K=3 (not shown in Table 2 and Fig. 3), the cluster comprising 15 putative hybrids was differentiated from the cluster comprising only *Pa. laevimanus* and *Pl. marinus* and the cluster comprising only *Pl. minutus* and *Pl. major*, but to a greater degree in the latter (F_ST_ = 0.244 *vs.* 0.400) (Table S2). Also at K=3, the two non-hybrid clusters were strongly differentiated from each other (F_ST_ = 0.727) (Table S2).

In RAD dataset 3 at K = 3 (not shown in Table 4 and Fig. 5), the cluster comprising individuals from the South Atlantic and Indian oceans (including one individual from the NWA) was approximately equally differentiated from clusters comprising individuals from the North Atlantic and Pacific oceans (F_ST_ = 0.086 and 0.079, respectively), which were more differentiated from each other than either was from the South Atlantic/Indian cluster (F_ST_ = 0.140) (Table S3)). Similar patterns were seen in the SNP-based Neighbor Net analyses of RAD dataset 3 (Fig. S7).

Patterns of genetic differentiation (F_ST_) among the regions designated in the AMOVA (Fig. 6; Table S4) were generally consistent with patterns (and associated F_ST_ values) among clusters identified by STRUCTURE and AWCLUST (Fig. 5; Table 4).

**Tables**

**Table S1.** Summary statistics of restriction-site associated DNA-sequencing (RAD*seq*) data processing.

| Putative species  designations  (Chace 1951; Poupin et al. 2005) | N | Raw Reads  (in millions) | | | Utilized Reads  (in millions) | | | Loci/Individual  (in millions) | | | Read Depth/Locus | |
| --- | --- | --- | --- | --- | --- | --- | --- | --- | --- | --- | --- | --- |
|  |  | Mean | Min | Max | Mean | Min | Max | Mean | Min | Max | Mean | s.d. |
| *Pachygrapsus laevimanus* | 6 | 0.71 | 0.46 | 0.86 | 0.51 | 0.37 | 0.67 | 0.11 | 0.07 | 0.13 | 4.6 | 28.7 |
| *Planes marinus* | 11 | 0.81 | 0.32 | 1.32 | 0.65 | 0.23 | 1.10 | 0.11 | 0.06 | 0.18 | 5.3 | 44.2 |
| *Planes minutus* | 52 | 1.04 | 0.33 | 1.56 | 0.89 | 0.21 | 1.34 | 0.12 | 0.04 | 0.17 | 6.4 | 67.2 |
| *Planes major* | 76 | 0.95 | 0.18 | 1.54 | 0.77 | 0.09 | 1.42 | 0.11 | 0.02 | 0.16 | 6.4 | 67.4 |
| Total | 145 | 0.90 | 0.31 | 1.34 | 0.72 | 0.21 | 1.15 | 0.11 | 0.04 | 0.16 | 6.0 | 51.0 |

**References**

Chace, F. A. (1951). The oceanic crabs of the genera *Planes* and *Pachygrapsus*. *Proceedings of the US National Museum, 101*, 65–103.

Poupin, J., Davie, P. J. F., & Cexus, J.C. (2005). A revision of the genus *Pachygrapsus* Randall, 1840 (Crustacea: Decapoda: Brachyura, Grapsidae), with special reference to the Southwest Pacific species. *Zootaxa, 1015*(1), 1–66. doi.org/10.11646/zootaxa.1015.1.1

**Table S2.** Pairwise comparison of genetic distance (F_ST_; below diagonal) and associated *P*-values (above diagonal), observed and expected heterozygosity and number of private alleles among clusters identified in RAD dataset 1.

|  | Clusters | |  |  | H_o_ (se) | H_e_ (se) | Pr |
| --- | --- | --- | --- | --- | --- | --- | --- |
| K = 2 (Fig. 3A) | 1 | 2 |  |  |  |  |  |
| Cluster 1 – *Pa. laevimanus* + *Pl. marinus +* 3 “RAD hybrids” | - | <0.0001 |  |  |  |  |  |
| Cluster 2 – *Pl. minutus* + *Pl. major* + 12 “RAD hybrids” | 0.683 | - |  |  |  |  |  |
|  |  | | |  |  |  |  |
| K = 3 (not shown in Fig. 3) | 1 | 2 | 3 |  |  |  |  |
| Cluster 1 – *Pa. laevimanus* + *Pl. marinus* | - | <0.0001 | <0.0001 |  |  |  |  |
| Cluster 2 – “RAD hybrids” | 0.244 | - | <0.0001 |  |  |  |  |
| Cluster 3 – *Pl. minutus* + *Pl. major* | 0.727 | 0.400 | - |  |  |  |  |
|  | | | |  |  |  |  |
| K = 4 (Fig. 3B) | 1 | 2 | 3 | 4 |  |  |  |
| Cluster 1 – *Pa. laevimanus* + *Pl. marinus* | - | <0.0001 | <0.0001 | <0.0001 | 0.068 (0.021) | 0.127 (0.032) | 14 |
| Cluster 2 – “RAD hybrids” | 0.244 | - | <0.0001 | <0.0001 | 0.253 (0.041) | 0.309 (0.032) | 0 |
| Cluster 3 – *Pl. minutus* | 0.728 | 0.358 | - | <0.0001 | 0.095 (0.030) | 0.102 (0.021) | 5 |
| Cluster 4 – *Pl. minutus* + *Pl. major* | 0.763 | 0.419 | 0.099 | - | 0.086 (0.032) | 0.090 (0.021) | 14 |
| Notes. H_o_, observed heterozygosity; H_e_, expected heterozygosity; Pr, number of private alleles | | | | | | | |

**Table S3.** Pairwise comparison of genetic distance (F_ST_; below diagonal) and associated *P*-values (above diagonal), observed and expected heterozygosity and number of private alleles among clusters identified in RAD dataset 3.

|  | Clusters | |  |  | H_o_ (se) | H_e_ (se) | Pr |
| --- | --- | --- | --- | --- | --- | --- | --- |
| K = 2 (Fig. 5A) | 1 | 2 |  |  |  |  |  |
| Cluster 1 – *Pl. minutus* (NWA,NEA,MED) ­+ *Pl. major* (SWA,SWI) | - | <0.0001 |  |  |  |  |  |
| Cluster 2 – *Pl. minutus* (NWA) + *Pl. major* (SEA,SWI,SEI,Pacific) | 0.122 | - |  |  |  |  |  |
|  |  | | |  |  |  |  |
| K = 3 (not shown in Fig. 5) | 1 | 2 | 3 |  |  |  |  |
| Cluster 1 – *Pl. minutus* (NWA,NEA,MED) | - | <0.0001 | <0.0001 |  |  |  |  |
| Cluster 2 – *Pl. minutus* (NWA) + *Pl. major* (SWA,SEA,SWI,SEI) | 0.086 | - | <0.0001 |  |  |  |  |
| Cluster 3 – *Pl. major* (Pacific) | 0.140 | 0.079 | - |  |  |  |  |
|  |  | | | |  |  |  |
| B (Fig. 5B) | 1 | 2 | 3 | 4 |  |  |  |
| Cluster 1 – *Pl. minutus* (NWA,NEA,MED) | - | <0.0001 | <0.0001 | <0.0001 | 0.188 (0.006) | 0.213 (0.004) | 29 |
| Cluster 2 – *Pl. minutus* (NWA) + *Pl. major* (SWA,SEA,SWI,SEI) | 0.086 | - | <0.0001 | <0.0001 | 0.179 (0.006) | 0.197 (0.005) | 2 |
| Cluster 3 – *Pl. major* (NWP,SWP,NCP,NEP) | 0.123 | 0.080 | - | <0.0001 | 0.191 (0.006) | 0.207 (0.004) | 0 |
| Cluster 4 – *Pl. major* (NWP,NCP,SCP,NEP,SEP) | 0.156 | 0.088 | 0.038 | - | 0.174 (0.006) | 0.198 (0.004) | 5 |
| Notes. H_o_, observed heterozygosity; H_e_, expected heterozygosity; Pr, number of private alleles | | | | | | | |

**Table S4.** Pairwise comparison of genetic distance (F_ST_; below diagonal) and associated *P*-values (above diagonal) among 11 ocean regions from AMOVA.

|  | MED | NWA | NEA | SA^†^ | IND^‡^ | NWP | SWP | NCP | SCP | NEP | SEP |
| --- | --- | --- | --- | --- | --- | --- | --- | --- | --- | --- | --- |
| Mediterranean Sea (MED) | – | 0.546 | 0.061 | 0.017 | 0.001 | <0.001 | 0.043 | <0.001 | 0.028 | <0.001 | <0.001 |
| Northwest Atlantic (NWA) | -0.010 | – | 0.838 | 0.007 | <0.001 | <0.001 | 0.008 | <0.001 | 0.001 | <0.001 | <0.001 |
| Northeast Atlantic (NEA) | 0.007 | -0.009 | – | 0.003 | <0.001 | <0.001 | 0.011 | <0.001 | 0.002 | <0.001 | <0.001 |
| South Atlantic (SA^a^) | 0.046 | 0.047 | 0.071 | – | 0.825 | <0.001 | 0.185 | 0.003 | 0.107 | 0.002 | 0.006 |
| Indian (IND^b^) | 0.040 | 0.085 | 0.095 | -0.037 | – | <0.001 | 0.013 | <0.001 | 0.010 | <0.001 | <0.001 |
| Northwest Pacific (NWP) | 0.127 | 0.133 | 0.132 | 0.076 | 0.088 | – | 0.007 | 0.005 | <0.001 | <0.001 | <0.001 |
| Southwest Pacific (SWP) | 0.151 | 0.128 | 0.151 | 0.093 | 0.093 | 0.090 | – | 0.008 | 0.196 | 0.018 | 0.009 |
| North Central Pacific (NCP) | 0.118 | 0.126 | 0.135 | 0.063 | 0.074 | 0.010 | 0.110 | – | 0.006 | 0.090 | <0.001 |
| South Central Pacific (SCP) | 0.198 | 0.224 | 0.230 | 0.138 | 0.156 | 0.109 | 0.188 | 0.081 | – | 0.012 | 0.017 |
| Northeast Pacific (NEP) | 0.135 | 0.140 | 0.150 | 0.075 | 0.077 | 0.032 | 0.154 | 0.005 | 0.071 | – | 0.471 |
| Southeast Pacific (SEP) | 0.142 | 0.158 | 0.166 | 0.082 | 0.105 | 0.109 | 0.150 | 0.015 | 0.066 | -0.005 | – |
| ^†^ Includes South West Atlantic (SWA) and South East Atlantic (SEA)  ^‡^ Includes South West Indian (SWI) and South East Indian (SEI). | | | | | | | | | | | |

**Figures**

**Figure S1.** Venn diagram showing the distribution of SNP loci among RAD datasets.

**Figure S2.** Histogram of haplotype divergence among loci in A) RAD dataset 1, B) RAD dataset 2, and C) RAD dataset 3.

**Figure S3.** Plots comparing statistical support for different numbers of populations (K) in STRUCTURE (likelihood [L[K]] and change in likelihood [delta K]) and AWCLUST (gap statistics) for A-C) RAD dataset 1, D-F) RAD dataset 2, and H-J) RAD dataset 3.


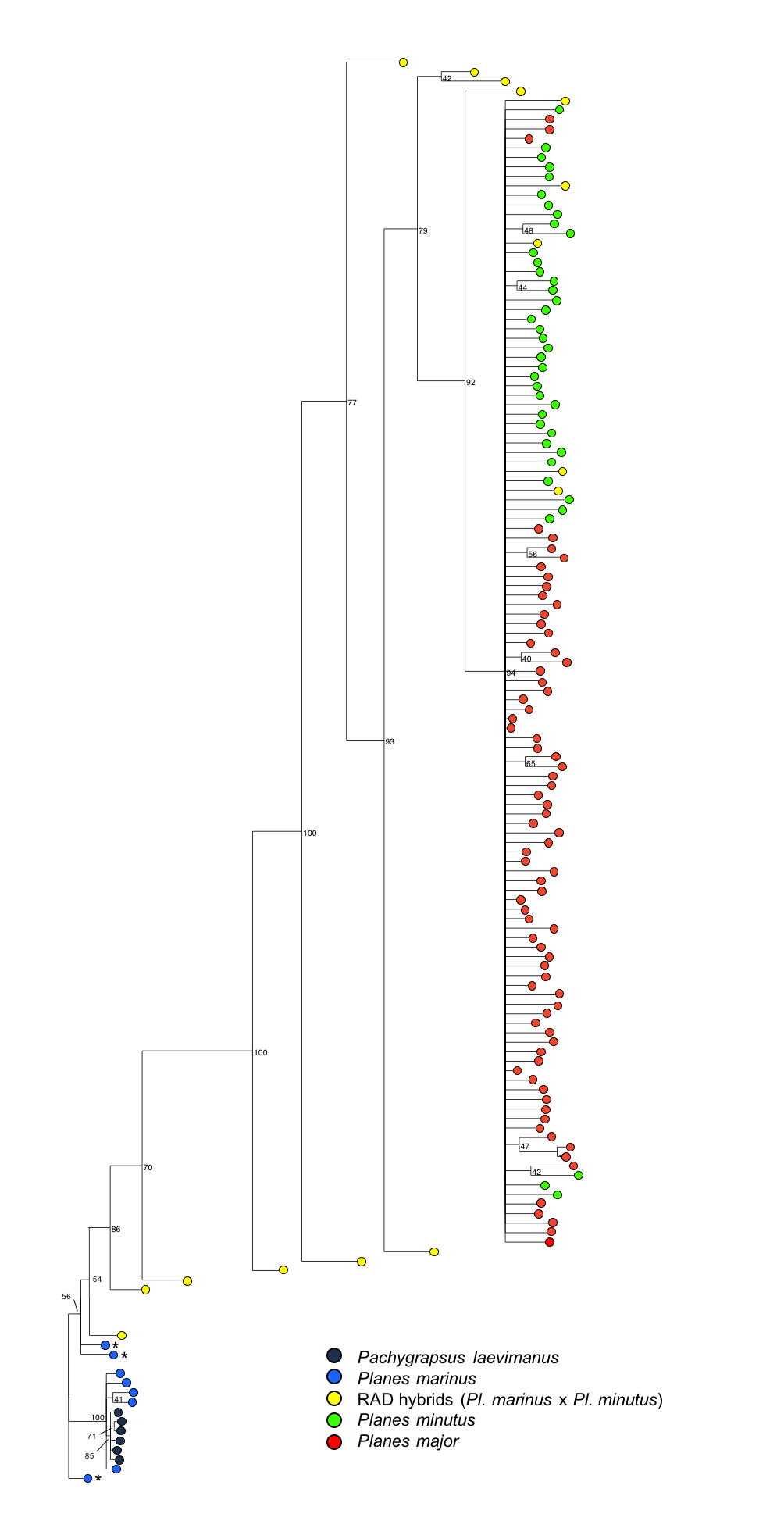


**Figure S4.** Maximum-likelihood phylogenetic analysis of RAD dataset 1 (N = 145 individuals; 6 *Pa. laevimanus*, 8 *Pl. marinus*, 15 “RAD hybrids”, 39 *Pl. minutus* and 77 *Pl. major*). Numbers at nodes indicate bootstrap support values and nodes with <40% bootstrap support are collapsed. Asterisks indicate additional putative hybrids from the Indian Ocean that were not identified in our STRUCTURE analysis at K=2.

**Figure S5.** Maximum-likelihood phylogenetic analysis of RAD dataset 2 (N = 14 individuals; 6 *Pa. laevimanus*, 8 *Pl. marinus*). Numbers at nodes indicate bootstrap support values.

**Figure S6.** Maximum-likelihood phylogenetic analysis of RAD dataset 3 (N = 116 individuals; 39 *Pl. minutus* and 77 *Pl. major*). Numbers at nodes indicate bootstrap support values.

**Figure S7.** SNP-based phylogenetic network for RAD dataset 3 (N = 116 individuals; 39 *Pl. minutus* and 77 *Pl. major*). Cluster labels are based on assignments from STRUCTURE and AWCLUST.
